# A ratio fluorescence method based on dual emissive gold nanoclusters for detection of biomolecules and metal ions

**DOI:** 10.1039/d2ra00131d

**Published:** 2022-04-20

**Authors:** Chenchen Kong, Yunjing Luo, Wei Zhang, Taifeng Lin, Zhen Na, Xuemei Liu, Ziqi Xie

**Affiliations:** Beijing Key Laboratory of Environmental and Viral Oncology, Faculty of Environment and Life, Beijing University of Technology No. 100, Pingleyuan, Chaoyang District Beijing 100124 China Luoyj@bjut.edu.cn

## Abstract

Gold nanoclusters have good biocompatibility and can be easily modified to improve their luminescence properties. In this study, we prepared a new type of dual-emitting gold nanoclusters (d-Au NCs) for discriminative detection of phenylalanine and Fe^3+^ with high selectivity and sensitivity. The fluorescence sensor which was synthesized without any further assembly or conjugation shows dual-emissions at 430 nm and 600 nm under a single excitation at 350 nm. Phenylalanine can turn on the red emission of the probe, while Fe^3+^ can turn on its yellow emission and turn off the red emission. By detecting a variety of amino acids and metal ions, d-Au NCs showed good selectivity to phenylalanine and Fe^3+^. Finally, this method was applied to determine phenylalanine and Fe^3+^ in lake water, human urine and milk, which has certain application prospects in the field of biology and environment.

## Abstract 2

Novel dual-emissive gold nanoclusters (d-Au NCs) were prepared and applied for the simultaneous detection of phenylalanine or Fe^3+^.

## Graphical abstract

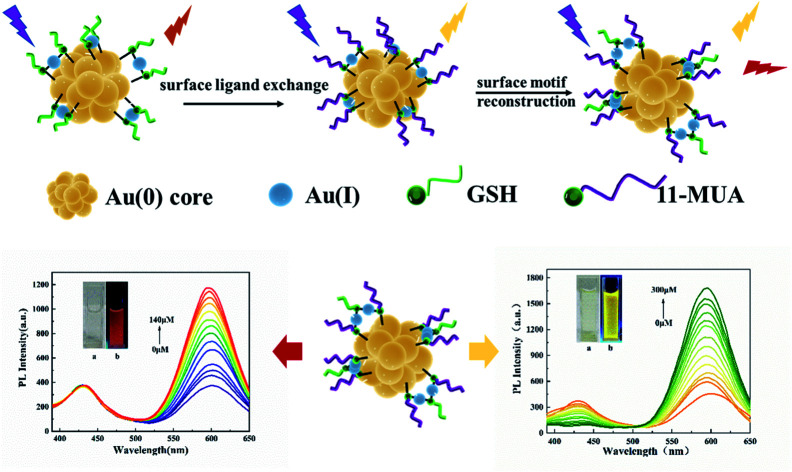

## 1. Introduction

As an emerging nanomaterial, gold nanoclusters have been widely used in bioimaging,^1,2^ optoelectronics^3,4^ and other fields.^5,6^ Especially in terms of fluorescent materials, they have become a substitute for many traditional fluorescent materials. As a new type of fluorescent cluster, Au NCs have better fluorescence performance than organic fluorescent molecules (such as semiconductor quantum dots), and have the advantages of mild synthesis conditions and environment friendliness.^7,8^ However, most reported Au NCs have a single emission wavelength. The single emission peak of gold nanoclusters has certain limitations for the detection and practical application of substances.^9,10^ The dual-emission gold nanoclusters not only retain the advantages of single-emission gold nanoclusters, but can also detect different substances at the same time, increasing the detection efficiency.^11,12^ In particular, the dual-emitting gold nanoclusters have the ability to self-calibrate,^13^ allowing more intuitive quantitative analysis.^14,15^ Therefore, the ratio fluorescence analysis method of Au NCs with two emission wavelengths has great research value.

Phenylalanine, as one of the essential amino acids of the human body, takes part in glucose metabolism and fat metabolism.^16,17^ The Guthrie bacterial growth inhibition assay^18,19^ is a common method to detect phenylalanine levels, but it takes a long time and requires rigorous testing conditions. Therefore, it is very important to develop a convenient and efficient method for the detection of phenylalanine. Fe^3+^ is an essential trace element in life activities,^20,21^ and it is an important redox pair in electron transport and various enzyme reactions.^22,23^ The lack and excess of iron ions will have some negative effects,^24,25^ Commonly used methods for iron ion determination, such as voltammetry, flame atomic absorption spectrophotometry (FAAS), inductively coupled plasma-atomic emission spectrometry (ICP-AES), are time-consuming and complicated.^26,27^ Therefore, a fast, low-cost, high-sensitivity and easy-to-operate method has attracted more and more attention, and the design of a fluorescent nanoprobe suitable for Fe^3+^ analysis still has value and significance.

The luminescence properties of metal clusters are affected by the synthesis method and ligand–metal charge transfer (LMCT) or ligand–metal–metal charge transfer (LMMCT). Doping other metal elements or ligands in the metal clusters can induce the LMCT effect and improve the luminescence properties. For the template method, the formation of Au NCs is achieved by reducing gold ions to the zero-valence state in the presence of thiol-terminated small molecules and a template-containing cavity. The selection of suitable templates is important for the synthesis of metal clusters that are stable and have special emission wavelengths. GSH-Au NCs with Au(0) core doped with 11-MUA can promote the LMCT effect to enable gold clusters with dual emission peaks. Herein, we directly synthesized dual-emissive gold nanoclusters (d-Au NCs) through surface ligand exchange and surface motif optimization and reconstruction, the synthesis process without additional assembly or conjugated. Synthesis of d-Au NCs was divided into two stages, the first stage is to synthesize GSH-Au NCs by template method, and the second stage is to exchange surface ligands and reconstruct surface motifs on the basis of GSH-Au NCs. Additionally, different techniques were used to characterize the synthesized d-Au NCs, in the presence of phenylalanine and Fe^3+^, there is a significant fluorescence turn-on effect.

## 2. Experimental

### 2.1 Chemicals and materials

Fluorescence measurements were performed on an F-4500 fluorescence spectrophotometer (Hitachi, Japan). UV–vis absorption spectra were recorded on a U-3010 spectrophotometer (Hitachi, Japan). The morphology and microstructure of gold nanocrystals were characterized by a transmission electron microscope (TEM) of JEOL F-200X (Japan). Fluorescence lifetime were performed on a steady state and transient state fluorescence spectrometer (Edinburgh Instruments FLA-1000). The constant temperature magnetic stirrer SH-3 (Beijing Jinke Development Company) was used to mix the solution.

### 2.2 Reagents

Glutathione (reduced form) (GSH) was obtained from Aladdin Chemical Reagent Co. (China). Chloroauric acid (HAuCl_4_·4H_2_O) was purchased from Sinopharm Chemical Reagent Co. (China). 11-mercaptoundecanoic acid (11-MUA), were obtained from Sigma Aldrich (Milwaukee, USA). Amino acids and metal salts were purchased from Sigma Aldrich. All reagents were of the highest available purity and of at least analytical grade.

### 2.3 Synthesis of d-Au NCs

The first stage is to synthesize glutathione-stabilized gold nanoclusters (GSH-Au NCs) which is yellow and transparent under visible light. Briefly, 300 μL of GSH (100 mM) and 1000 μL freshly prepared aqueous solution of HAuCl_4_ (20 mM) were mixed with 8700 μL ultrapure water. The entire reaction was prepared under dark condition. After the mixture was continuously stirred at 70 °C for 24 hours, a yellow solution was obtained as GSH-Au NCs. The prepared solution can be stored at 4 °C for further study.

Then, prepare d-Au NCs by reacting with 11-mercaptoalkanoic acid (11-MUA) on the basis of GHS-Au NCs. 200 μL of PBS buffer solution (pH = 9) was added to 1 mL of GHS-Au NCs, mixed with 200 μL of 11-MUA (100 mM) dissolved in ethanol, and finally 140 μL of NaOH (0.5 M) was added to make the mixed solution alkaline. The mixed liquid was stirred at room temperature for 40 hours. The settled solution obtained after completion is d-Au NCs. All glassware used in the preparation process was soaked in piranha solution for 30 minutes and rinsed with distilled water.

The prepared d-Au NCs was detected in the full wavelength range on an F-4500 fluorescence spectrophotometer. Select the excitation wavelength for the strongest fluorescence emission peak.

### 2.4 Fluorescent detection of phenylalanine and Fe^3+^

Detect phenylalanine and iron ions with synthetic d-Au NCs, before detection, 0.5 M HCl was added to d-Au NCs to make the pH of the solution close to 10.

For detection of phenylalanine, phenylalanine solution with a concentration of 0–150 μM was added to d-Au NCs separately. Under 350 nm excitation, fluorescence spectra were recorded in the wavelength range of 390 nm to 650 nm. For detection of Fe^3+^, Fe^3+^ solutions with different concentrations (0–300 μM) were separately added into the d-Au NCs solution. Similarly, under the excitation of 350 nm, the fluorescence spectra were recorded in the wavelength range of 390 nm to 650 nm.

### 2.5 Selectivity measurement

150 μM of various kinds of amino acids were added to d-Au NCs instead of phenylalanine to evaluate the selectivity to phenylalanine. The selectivity of the sensing method was evaluated by comparing the ratio of the fluorescence peaks *I*_600_ to *I*_430_. To further examine the selectivity of d-Au NCs complexes toward Fe^3+^, different metal ions will be added to d-Au NCs. By comparing the fluorescence intensity of each metal ion at a ratio of *I*_600_ and *I*_430_ to evaluate the selectivity of Fe^3+^.

## 3. Results and discussion

### 3.1 Characterization of GSH-Au NCs and d-Au NCs

**Scheme 1:**
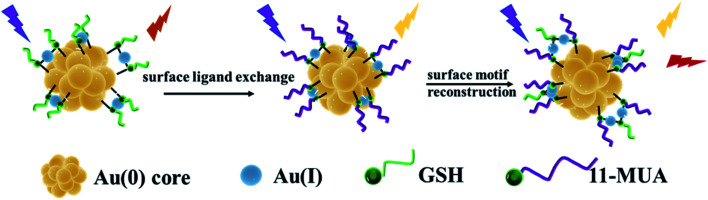
Dual-emissive gold nanoclusters.

**Fig. 1:**
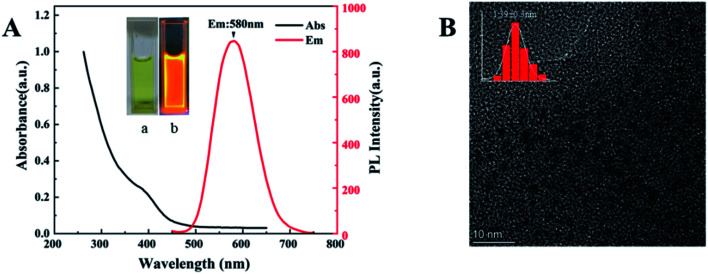
(A) PL and UV-vis absorption spectra of GSH-Au NCs. Inset: the photographs of GSH-Au NCs solution (a) in ambient light and (b) under 365 nm UV light. (B)TEM image of GSH-Au NCs.

Dual-emission Au NCs probes are mainly completed in two steps, the synthetic route is shown in Scheme 1. In the process of preparing GSH-Au NCs, the sulfhydryl end of glutathione provides an appropriate protection for the metallic core through the Au–S bonding.^28,29^ The optical characteristics of GSH-Au NCs are shown in Fig. 1. In the UV-vis spectra, there is a shoulder peak at 400 nm, and the excitation range is 300–400 nm. In our experiment, the aggregation-induced emission mechanism causes strong emission of GSH-Au NCs. When excitation is 395 nm, the fluorescence emission peak is at 580 nm.

The prepared GSH-Au NCs are light yellow under sunlight and bright orange under ultraviolet light (Fig. 1A), in a dark environment at 4 °C, it can be stored for more than 4 months without changes in properties. TEM image evidences an ultra-small size with uniformly dispersed particles for the GSH-Au NCs (Fig. 1B), and the average diameter is 1.39 nm.

**Fig. 2:**
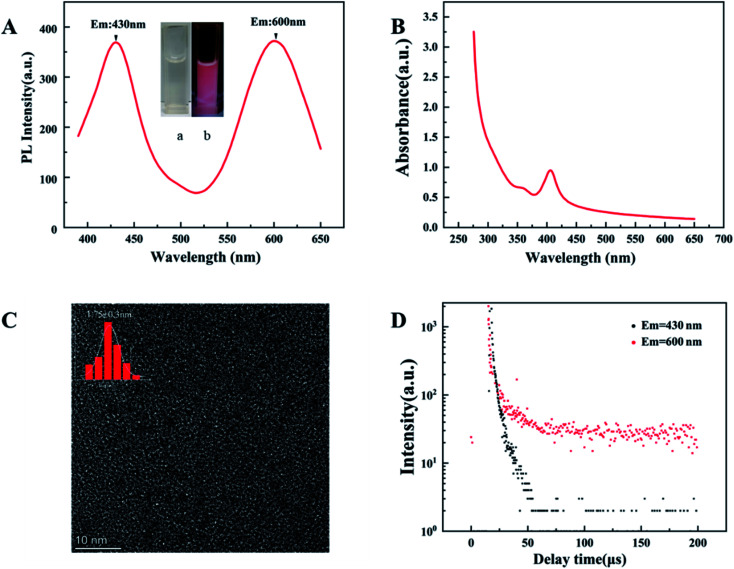
(A) PL spectrum of d-Au NCs (*λ*_ex_ = 350 nm). Inset: the photographs of d-Au NCs solution (a) in ambient light and (b) under 365 nm UV light. (B) UV-vis absorption spectra of d-Au NCs. (C) TEM image of d-Au NCs. (D) PL spectra of d-Au NCs after storage for different times.

The second step is to synthesize d-Au NCs through surface motifs and reconstruction on the basis of GSH-Au NCs. The prepared d-Au NCs are transparent white under sunlight and pink under ultraviolet light (Fig. 2A). The d-Au NCs can be stored in a dark environment at room temperature for more than 4 months without changes in properties. The optical properties show that d-Au NCs have two emission peaks at 430 nm and 600 nm when excited at 350 nm (Fig. 2A). In the UV-vis spectrum, there are two shoulder peak at 360 nm and 460 nm (Fig. 2B). The TEM image shows that the size of d-Au NCs 1.75 ± 0.3 nm, and the distribution is uniform (Fig. 2C). In addition, the average lifetime of the emission peak at 430 nm of d-Au is 6 μs, and at 600 nm emission is 10 μs (Fig. 2D).

After surface ligand–metal charge transfer effect and surface motif reconstruction, d-Au NCs produced two emission peaks. During the surface ligand exchange process, from the Au(i)–11-MUA motif to the Au(0) nuclear ligand–metal charge transfer (LMCT) effect,^30^ a new fluorescence emission peak was generated at 430 nm. After the rapid completion of the ligand exchange, in order to keep the system at the lowest surface energy to reach an equilibrium state, the GSH free in the solution will be redistributed and bound to the Au NCs along with the optimization of the surface ligands. 11-MUA and GSH are recombined on the surface of d-Au NCs through Au–S bond. Compared with the surface ligand exchange in the previous stage, the recombination of the surface ligands in the second part takes up more synthesis time. The surface ligand recombination transfers the ligand–metal charge from the surface Au(i)-GSH motif to the Au(0) core, which further leads to the second fluorescence emission peak of d-Au NCs at 600 nm.

### 3.2 Interaction with phenylalanine and Fe^3+^

**Fig. 3:**
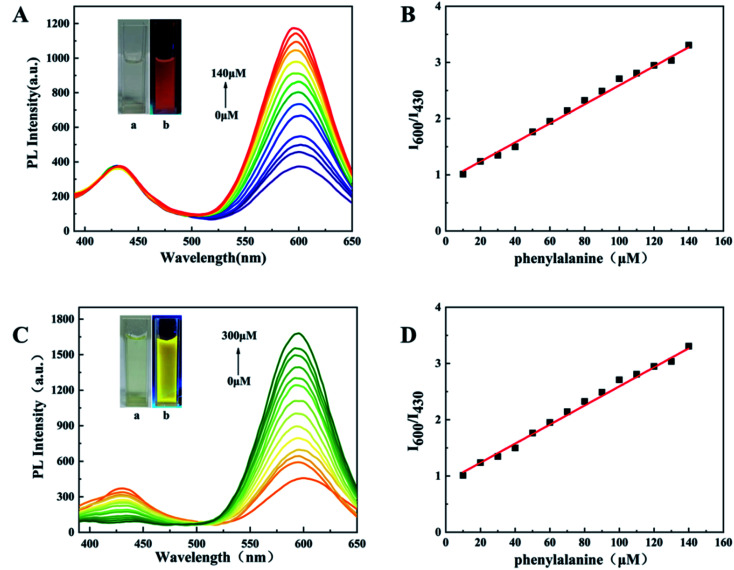
(A) PL spectra of d-Au NCs with addition of different concentrations of phenylalanine (0–140 μM). Inset: UV light-irradiated d-Au NCs solutions after addition of different concentrations of phenylalanine. (B) The linear relationship between *I*_600_/*I*_430_ and the phenylalanine concentration. (C) PL spectra of d-Au NCs with addition of different concentrations of Fe^3+^ (0–300 μM). Inset: UV light-irradiated d-Au NCs solutions after addition of different concentrations of Fe^3+^. (D) The linear relationship between *I*_600_/*I*_430_ and the Fe^3+^concentration.

The interaction of d-Au NCs with phenylalanine can produce a significant fluorescence turn-on response (Fig. 3A). As the concentration of phenylalanine increases (0–140 μM), under the excitation of 350 nm, the emission intensity at 600 nm gradually increases to about 3 times the original intensity, while the emission intensity at 430 nm remains consistent.

The color of mixed solution gradually turns from pink to red under UV light. The ratio of fluorescence intensity at 600 nm and 430 nm has a linear relationship with the concentration of phenylalanine, and the detection limit is 3.9 μM (Fig. 3B). This indicates that d-Au NCs can be used as a ratio probe for the quantification of phenylalanine and the detection process can be completed within 5 minutes. In addition, a variety of other amino acids have been tested, but only phenylalanine has good selectivity.

D-Au NCs not only has a good fluorescence effect on phenylalanine, but also a good fluorescence response to Fe^3+^. After adding Fe^3+^ (0–300 μM) to d-Au NCs, the emission intensity at 600 nm gradually increased, while the emission intensity at 430 nm gradually decreased, which was completely different from the phenylalanine effect. The solution gradually changed from pink to yellow under ultraviolet light (Fig. 3C). When the detection range of Fe^3+^ is 0–300 μM, the ratio of PL intensities at 430 nm and 600 nm has a good linear relationship with its concentration, and the detection limit is 2.04 μM (Fig. 3D). The detection of iron ions can also be completed within five minutes, and through the detection of different metal ions, d-Au NCs has better detection to Fe^3+^.

**Fig. 4:**
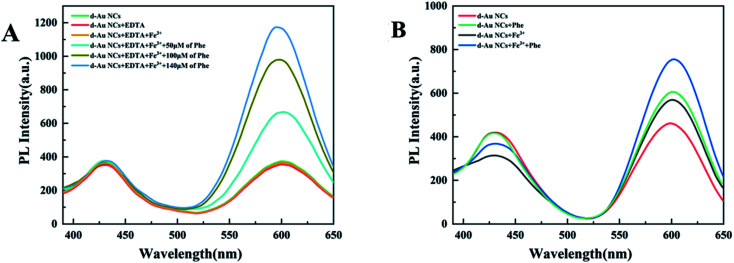
(A) Experimental verification for eliminating Fe^3+^ interference by adding EDTA in detection of phenylalanine. (B) PL spectra of d-Au NCs with simultaneous addition of Phe and Fe^3+^.

In further experiments, phenylalanine and Fe^3+^ were simultaneously added to d-Au NCs to achieve simultaneous detection. The effect of Fe^3+^ was eliminated by adding EDTA and not adding EDTA respectively. Fig. 4 shows that d-Au NCs can detect both phenylalanine and Fe^3+^ in the solution.

According to the previous experiment, d-Au NCs has a strong fluorescence reaction to phenylalanine and Fe^3+^. The main reason is related to the structure of d-Au NCs itself. The enhancement effect of the emission peak is due to the dedication of free electrons to the metal core. Meanwhile, it is the electron-rich atoms or groups on the surface ligands of the gold clusters that promote this behavior of contribution.

Phenylalanine has a good opening effect on the emission peak at 600 nm, but has no effect on the emission peak at 430 nm. This phenomenon is caused by the interaction between phenylalanine and surface motifs on d-Au NCs. Phenylalanine promotes the charge transfer effect from the Au(i)-GSH motif to the Au(0) core, thus enhancing the fluorescence emission intensity at 600 nm. Since the sulfhydryl and carboxyl groups of 11-MUA are far apart, the binding between the carboxyl group and phenylalanine of 11-MUA has basically no effect on the charge transfer from Au(i)–11MUA motif to Au(0) core. Therefore, the fluorescence peak is basically no change at 430 nm.

After adding Fe^3+^, the emission intensity at 430 nm decreases, the reason for this phenomenon is that Fe^3+^ has a strong electron-withdrawing ability. The interaction between Fe^3+^ and Au(i)–11MUA motif on the surface. The strong coordination of Fe^3+^ promotes the transfer of the Au(i)–11MUA motif to the Au(0). The chelation effects of the Fe^3+^ make them with high affinity to d-Au NCs, which can promote the charge transfer of Au(i)-GSH motif to Au(0) nucleus and lead to the enhancement of 600 nm emission peaks.

### 3.3 Selectivity study

**Fig. 5:**
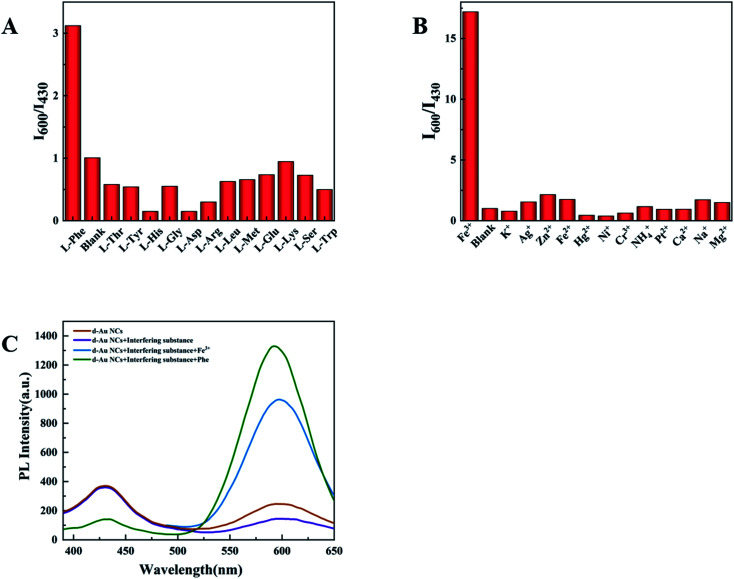
(A) *I*_600_/*I*_430_ values of d-Au NCs in the presence of different kinds of amino acids with a concentration of 140 μM. (B) *I*_600_/*I*_430_ values of d-Au NCs in the presence of different kinds of metal ions with a concentration of 300 μM. (C) PL spectra of Phe and Fe^3+^ added to a mixture of amino acids and ions.

In this experiment, some substances were selected to test the selectivity of d-Au NCs to phenylalanine and Fe^3+^, such as biomolecules(l-threonine, l-tyrosine, l-histidine, l-glycine, l-asparaginase, l-arginine, l-leucine, l-methionine, l-glutamine, l-lysine, l-serine, l-tryptophan) and metal ions (K^+^, Ag^+^, Zn^2+^, Fe^2+^, Hg^2+^, Ni^+^, Cr^3+^, NH_4_^+^, Pt^2+^, Ca^2+^, Na^+^, Mg^2+^). As mentioned earlier, after adding phenylalanine or Fe^3+^, the fluorescence intensity of d-Au NCs has been significantly enhanced. When other detection materials were added, the ratio of the fluorescence peak at 600 nm to 430 nm did not increase significantly. It shows that d-Au NCs can be used as a ratio probe for the quantification of phenylalanine and Fe^3+^ (Fig. 5).

### 3.4 Optimization of the experimental variables

**Fig. 6:**
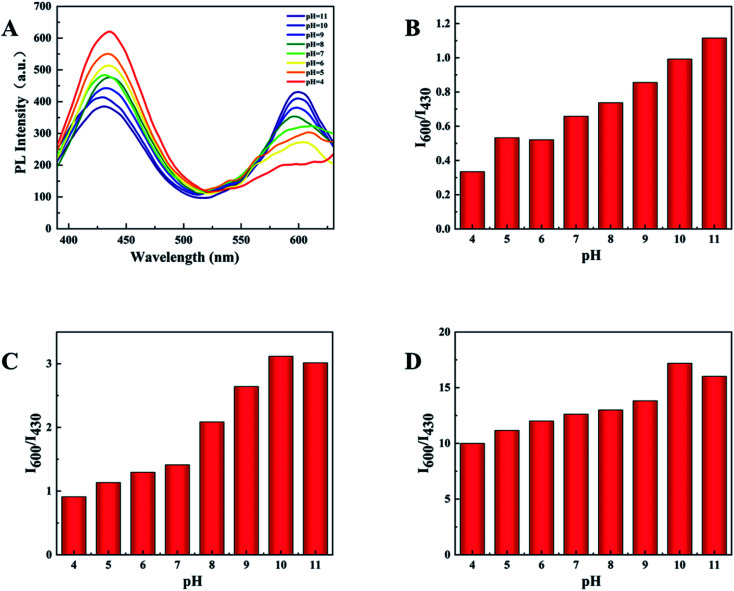
(A) PL spectra of d-Au NCs at different pH (4–11). (B) *I*_600_/*I*_430_ values of d-Au NCs at different pH (4–11). (C) *I*_600_/*I*_430_ values of d-Au NCs with Phe at different pH. (D) *I*_600_/*I*_430_ values of d-Au NCs with Fe^3+^ at different pH.

The sensitivity of the sensor is determined by the ratio of the fluorescence intensity at 600 nm and 430 nm (*I*_600_/*I*_430_). In order to obtain the highest sensitivity and the most intuitive peak change, pH and temperature in the experiment were explored and optimized. The experiment explored the influence of different pH (4–11) on the two emission peaks (Fig. 6A). When the pH is 10, *I*_600_/*I*_430_ reaches the maximum after adding the test substance, and the ratio of *I*_600_/*I*_430_ of d-Au NCs is closest to 1(Fig. 6B), which is more convenient for comparison before and after the test. And in the presence of phenylalanine and Fe^3+^, the maximum *I*_600_/*I*_430_ ratio was obtained at pH 10.

**Fig. 7:**
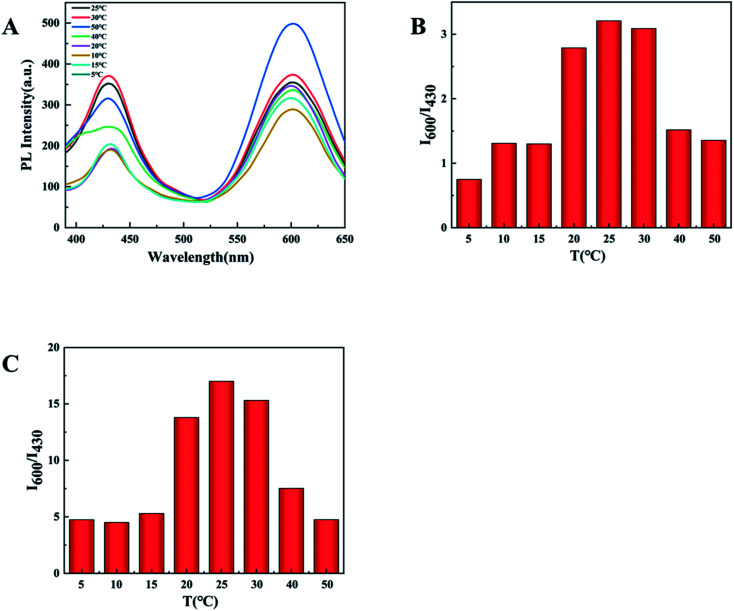
(A) PL spectra of d-Au NCs at different temperature (5–50 °C). (B) *I*_600_/*I*_430_ values of d-Au NCs with Phe at different temperature. (C) *I*_600_/*I*_430_ values of d-Au NCs with Fe^3+^ at different temperature.

Furthermore, we also tested the influence of temperature within 5–50 °C on the detection. It can be clearly seen from the Fig. 7 that the optimal fluorescence spectrum can be obtained at 25 °C, indicating that the sensor system is stable at room temperature. In the future testing process, we choose to carry out experiments at room temperature (23–28 °C).

### 3.5 Fluorometric detection of phenylalanine and Fe^3+^in samples

In order to test the feasibility of phenylalanine and Fe^3+^ in actual samples (lake water, human urine and milk), we add phenylalanine and Fe^3+^ (5 μM) to the samples respectively, and analysed each samples as the described procedure earlier. The recoveries and relative standard deviations (RSD) for the detection of phenylalanine and Fe^3+^ in lake water, human urine and milk are listed in Table 1. The results show that the recoveries of phenylalanine are in the range of 98.4–99.2% and the recoveries of Fe^3+^ are in the range of 98.2–102.2%. The RSD was below 3% for three replicate measurements. This method shows satisfactory recovery and low RSD, and the proposed sensor has a good reliability for potential applications.

**Table 1:** Results for the phenylalanine and Fe^3+^ determination in sample

	Sample	Add (μM)	Found (μM)	Recovery (%)	RSD (*n* = 3, %)
Phenylalanine	Lake	0.00	ND	—	—
		5.00	4.96	99.2	1.3
	Urine	0.00	ND	—	—
		5.00	4.92	98.4	1.2
	Milk	0.00	ND	—	—
		5.00	4.93	98.6	1.5
Fe^3+^	Lake	0.00	ND	—	—
		5.00	5.11	102.2	2.6
	Urine	0.00	ND	—	—
		5.00	4.97	99.4	1.5
	Milk	0.00	ND	—	—
		5.00	4.91	98.2	1.3

## 4. Conclusion

In summary, we successfully synthesized dual-emissive gold nanoclusters without additional assembly or conjugation. The fluorescence lifetime and nanostructure of d-Au NCs have been studied and confirmed through steady-state/transient fluorescence spectrometer and high-resolution transmission electron microscopy. The average decay times of the d-Au NCs were 5.8 μs for 430 nm emission and 10 μs for 600 nm emission. The particle size of d-Au NCs is uniform, and the diameter is 1.75 ± 0.3 nm. Based on the LMCT effects generated by surface motif reconstruction, d-Au NCs have two emission peaks at the same time. Different from the detection of a single species by a single emission peak, d-Au NCs can realize the simultaneous exploration of two different species. The prepared gold nanoclusters can complete the detection of phenylalanine and Fe^3+^ within five minutes at room temperature. Under ultraviolet light irradiation, the naked eye can directly monitor the detection effect through the change of color. The prepared fluorescence sensor offered high selectivity for phenylalanine and Fe^3+^ over other amino acids. D-Au NCs have higher detection efficiency than single emission metal clusters. This detection method can provide faster and easier multi-substance detection compared to traditional detection methods. Therefore, we conceive that this study will encourage the further development of Au NCs for simultaneous detection of multiple species.

## Author contribution

Chenchen Kong: design the experimental process, synthetic polymers, write and revise the manuscript. Yunjing Luo: design the experimental process, guide experiment, revise the manuscript, supervision. Wei Zhang: analyze and discuss the results. Taifeng Lin: analyze and discuss the results. Zhen Na: collect and organize literature. Xuemei Liu: revise the manuscript. Ziqi Xie: revise the manuscript.

## Conflicts of interest

There are no conflicts to declare.

## Supplementary Material
